# Performance-Enhancing Effects of Inhaled Medications: Implications for Heart, Muscle Function, and Doping Detection in Athletes

**DOI:** 10.3390/jfmk10040462

**Published:** 2025-11-26

**Authors:** Riccardo Cricco, Andrea Segreti, Emanuele Stirpe, Aurora Ferro, Martina Ciancio, Flavia Cipriani, Chiara Fossati, Gian Paolo Ussia, Fabio Pigozzi, Francesco Grigioni

**Affiliations:** 1 Department of Cardiovascular Sciences, Fondazione Policlinico Universitario Campus Bio-Medico, Via Alvaro del Portillo 200, 00128 Rome, Italy; 2 Unit of Cardiovascular Sciences, Department of Medicine and Surgery, Università Campus Bio-Medico di Roma, 00128 Rome, Italy; 3 Department of Respiratory Disease, Hospital of Bolzano (SABES-ASDAA), Via Lorenz Böhler 5, 39100 Bolzano, Italy; 4 Department of Movement, Human and Health Sciences, University of Rome “Foro Italico”, Piazza Lauro de Bosis 15, 00135 Rome, Italy

**Keywords:** inhaled medications, sport medicine, doping, athletes, muscle, cardiovascular system

## Abstract

Inhaled medications, commonly prescribed for respiratory conditions such as asthma and exercise-induced bronchoconstriction, are increasingly scrutinized in sports medicine due to their potential performance-enhancing effects. Bronchodilators, in particular, may improve lung function, increase oxygen delivery, and influence muscle contractility, potentially enhancing athletic performance. However, supratherapeutic use raises concerns about cardiovascular risks, including tachyarrhythmias and altered autonomic balance, as well as muscle hypertrophy and sprint capacity gains. These effects blur the line between therapeutic use and doping, creating challenges for fair competition. This review explores the mechanisms by which inhaled drugs affect the cardiovascular and muscular systems, summarizes notable doping cases, and evaluates current detection methods. Despite regulatory thresholds established by the World Anti-Doping Agency, assay interpretation remains complicated by inter-individual variability, short drug half-lives, and enantiomeric differences. Addressing these gaps requires refined pharmacokinetic modeling, enantioselective assays, and metabolomic fingerprinting to safeguard both athlete health and the integrity of sport.

## 1. Introduction

### 1.1. Overview of Inhaled Medication Use Among Athletes and Prevalence

Inhaled medications, particularly β_2_-adrenergic agonists and corticosteroids, are commonly used for the management of respiratory conditions such as asthma, exercise-induced bronchoconstriction (EIB), and chronic obstructive pulmonary disease (COPD). Asthma is a highly prevalent disease in the general population, affecting about 6.6% of adults, 9.1% among children and 11.0% among adolescents [1]. Although its prevalence is lower in low-income countries compared to high-income countries, the associated burden is more severe [2]. Asthma prevalence is reported to be higher in athletes. Data suggest that up to 50% of endurance athletes report some degree of asthma and EIB [3]. Aquatic and winter-based sporting disciplines are also particularly associated with a higher percentage of athletes suffering from lower airway dysfunction [4,5].

The pathogenesis of EIB in athletes is multifactorial and influenced by sport type, training environment, genetics, and individual susceptibilities. High-intensity exercise often requires predominantly oral breathing, bypassing the natural filter of the upper airways and exposing the lower airways to physical, thermal, and chemical stress. In susceptible individuals, this can trigger airway dysfunction and bronchoconstriction, leading to respiratory symptoms such as cough, wheezing, and dyspnoea [6,7].

For athletes suffering from asthma or EIB, the recommended treatment mirrors that of the general population and is based on inhaled medications, including inhaled corticosteroids (ICS), short-acting beta-agonists (SABA), leukotriene receptor antagonists (LTRA), and mast cell stabilizers (MCSA) [4,8].

### 1.2. Potential for Misuse Among Athletes

According to the World Anti-Doping Code, a substance or method is included on the Prohibited List if it meets at least two of the following criteria: it has the potential to enhance sport performance, it represents an actual or potential health risk to the athlete, or its use violates the spirit of sport. Substances or methods that can mask the use of other prohibited substances or methods are also included [9].

While inhaled drugs are therapeutic for managing asthma and EIB, their potential misuse to enhance performance has raised concerns. Inhaled β_2_-agonists are the most extensively studied: salbutamol, formoterol, and terbutaline are permitted under specific conditions. However, some studies show these substances may enhance muscle strength and sprint performance, suggesting possible ergogenic effects [10,11]. Several high-profile doping cases involving β_2_-agonists have highlighted the challenge of distinguishing legitimate therapeutic use from potential misuse [12,13,14].

These medications induce bronchodilation, improving airflow and oxygen delivery during exertion, though this effect may be less significant than the direct muscular effects [3]. According to WADA, systemic corticosteroids are prohibited in-competition because of their potential performance-enhancing effects. In contrast, inhaled corticosteroids (ICS) are permitted both in- and out-of-competition, as evidence for any ergogenic effect is lacking [15].

This has led to debate in the scientific community regarding the thresholds at which therapeutic agents may confer a competitive advantage.

## 2. Mechanisms of Action of Inhaled Medications

### 2.1. Bronchodilators

Inhaled bronchodilators are the cornerstone of the symptomatic treatment of several respiratory diseases, particularly asthma, EIB and COPD [16]. They act primarily inducing relaxation of bronchial smooth muscle, thereby relieving airway obstruction and improving airflow. They are classified by mechanism of action and receptor target into β_2_-adrenoceptor agonists, anticholinergics, and theophylline.

#### 2.1.1. β_2_-Agonists

They are the ones used most frequently in both asthma and COPD management. Based on their pharmacodynamics, they are classified into Short-Acting Beta Agonists (SABAs) and Long-Acting Beta Agonists (LABAs).

- SABAs: Provide quick relief of symptoms and are typically administered in case of exacerbation, but are not recommended for chronic treatment. Examples include salbutamol and terbutaline [17,18].
- LABAs: Commonly used as maintenance therapy. Examples include formoterol and salmeterol [17].

These drugs activate β_2_-adrenergic receptors on airway smooth muscle cells. The β_2_-adrenergic receptor is a G-protein-coupled receptor composed of a heterotrimer (Gαs, Gβ, Gγ). When a β_2_-agonist binds this receptor, the heterotrimer dissociates into a Gαs monomer and a Gβγ dimer. Gαs undergoes a conformational change and binds to adenylate cyclase, the enzyme that catalyzes the conversion of adenosine triphosphate (ATP) into cyclic adenosine monophosphate (cAMP), an intracellular second messenger [19].

Elevated cAMP activates protein kinase A (PKA), which phosphorylates downstream targets. One important PKA effect is activation of myosin phosphatase, which dephosphorylates the myosin light chain (MLC). Dephosphorylated MLC cannot bind actin, reducing smooth muscle contraction and leading to relaxation.

Additionally, the Gβγ dimer activates potassium (K^+^) channels, causing K^+^ efflux, hyperpolarization, and reduced calcium mobilization, further inhibiting contraction. cAMP also activates Epac, which downregulates Rho and contributes to airway smooth muscle relaxation [20,21].

#### 2.1.2. Anticholinergics

Like β_2_-agonists, they are classified by duration of action into

- Short-Acting Muscarinic Antagonists (SAMAs): sometimes used with SABAs in acute settings (e.g., ipratropium bromide).
- Long-Acting Muscarinic Antagonists (LAMAs): such as tiotropium, which is rarely used in asthma but more commonly employed in COPD.

Their mechanism is blocking muscarinic receptors on airway smooth muscle and mucous glands. M3 receptors are the most relevant on bronchial smooth muscle, but M2 autoreceptor dysfunction and increased M1 activation also contribute to bronchoconstriction, mucus secretion, inflammation, and airway remodeling [22,23].

Blocking acetylcholine reduces contractility by preventing activation of protein kinase C (PKC) and inositol trisphosphate (IP3) signaling. Normally, PKC inhibits myosin light chain phosphatase, and IP3 triggers Ca^2+^ release from the endoplasmic reticulum. Both mechanisms increase contraction; blocking them promotes relaxation [24].

Combining a LAMA with a LABA offers complementary mechanisms: LAMA blocks parasympathetic-induced constriction, while LABA stimulates β_2_-receptors to enhance cAMP-mediated relaxation. This dual approach has been shown to improve lung function and reduce exacerbations in chronic respiratory conditions [25].

Although anticholinergic bronchodilators improve airflow and ventilatory capacity, there is currently no convincing evidence that they provide meaningful ergogenic benefits in healthy or athletic populations. Accordingly, anticholinergics are not included on the WADA Prohibited List.

#### 2.1.3. Theophylline

Theophylline is a methilxanthine and a minor metabolite of caffeine, currently used for a variety of respiratory conditions including EIB. Its mechanism of action involves non-selective inhibition of phosphodiesterase, leading to increased intracellular cyclic AMP, as well as antagonism of adenosine receptors, which contributes to bronchodilation and mild central nervous system stimulation [26]. Beyond its respiratory applications, theophylline has been investigated for potential ergogenic properties. Although it is not currently included in the WADA Prohibited List, theophylline was classified as a forbidden substance for several years due to its central nervous system–stimulating properties, for example, by the Flemish Government in 1991. As a methylxanthine structurally related to caffeine, it may enhance physical performance through adenosine receptor antagonism, leading to increased alertness, reduced perception of effort, and improved ventilatory efficiency. Evidence indicates that theophylline can exert mild performance-enhancing effects, particularly in endurance-based activities, though the studies results are inconsistent contradicting results and/or insufficient data [27].

### 2.2. Corticosteroids and Other Inhaled Drugs

Bronchial asthma is characterized by persistent inflammation even during remission. ICS such as beclomethasone, budesonide, and fluticasone act by reducing airway inflammation, improving airflow, controlling symptoms, and preventing exacerbations [8]. Unlike systemic corticosteroids, ICS have minimal systemic absorption at therapeutic doses, reducing side effects [28]. They are often combined with LABAs or used in triple therapy (LABA/LAMA/ICS) in patients with frequent COPD exacerbations or hyper-eosinophilia, and in asthma uncontrolled by monotherapy. The combination of LABAs and ICS plays a critical role in chronic asthma management, since LABA monotherapy has been associated with loss of effectiveness over time (tachyphylaxis) due to down-regulation or uncoupling of β_2_-adrenergic receptors on airway smooth muscle and inflammatory cells. ICS contribute to restoring β_2_-receptor function by increasing receptor expression and reversing agonist-induced desensitization, thereby enhancing the anti-inflammatory and bronchodilator responses of the combination therapy.

Mechanism: ICS bind to intracellular glucocorticoid receptors (GRs) in epithelial and immune cells. The drug–receptor complex translocates to the nucleus, where it interacts with glucocorticoid response elements (GREs). This leads to upregulation of anti-inflammatory genes (e.g., lipocortin-1, which inhibits phospholipase A2), and downregulation of pro-inflammatory genes (e.g., IL-4, IL-5, TNF-α, COX-2). Consequences: reduced recruitment of inflammatory cells, decreased mucus production, reduced vascular permeability, and induction of eosinophil apoptosis, limiting fibrosis and airway remodelling [29,30,31].

In patients with asthma, ICS help by suppressing the underlying airway inflammation and reducing hyper-responsiveness, while LABAs act on different pathophysiological pathways, not only by bronchodilation, but also by inhibiting mast cell mediator release, reducing plasma exudation and possibly decreasing sensory nerve activation. Moreover, there are positive molecular interactions: corticosteroids up-regulate β_2_-receptor gene expression (thus counteracting potential receptor down-regulation from long-term β_2_-agonist use), and β_2_-agonists may enhance corticosteroid efficacy by increasing nuclear localization of glucocorticoid receptors and amplifying anti-inflammatory effects [32].

### 2.3. How These Medications Improve Respiratory Function and Overall Performance

Bronchodilators improve pulmonary function by relaxing smooth muscle via increased cAMP, causing bronchodilation and reduced airflow resistance. This enhances ventilation, oxygen uptake, and CO_2_ elimination [33]. This condition of improved distribution of airflow throughout the lungs translates into an increase in Forced Expiratory Volume in 1 s (FEV_1_) and Peak Expiratory Flow (PEF) relative to baseline, even in non-asthmatic individuals [34]. Also ICS have shown to improve lung function, symptoms, and quality of life and to reduce exacerbations in both COPD and asthma [35]. Consequently, oxygen delivery to the muscles is optimized, which can delay fatigue and improve endurance. This could be particularly helpful during physical exertion when greater skeletal muscle performance is requested, especially during aerobic activities. However, some studies have shown that the positive effect on FEV1 induced by β_2_-agonists administration, is not accompanied by an increase in aerobic performance outcomes, such as maximum oxygen uptake (VO 2max), time trial performance, time to exhaustion and minute ventilation at peak exercise [36,37].

There is evidence of a clear benefit of inhaled medications on overall performance in people affected by chronic respiratory diseases [21,34]. However, several studies have questioned the performance-enhancing effect of inhaled drugs on healthy people, especially athletes [38,39].

Non-asthmatic athletes may also experience EIB, particularly under extreme environmental conditions [40,41]. Commonly, short-acting bronchodilators are used 15–30 min before exercise to prevent bronchoconstriction onset. However, recent studies and recommendations discourage the use of SABA as a sole treatment [4,8].

The assumption that inhaled medications have the potential to improve physical performance, resulting in an unfair competitive advantage when taken by healthy athletes brought the anti-doping agencies to regulate their administration. In athletes suffering from asthma and EIB, however, use of inhaled medication is fundamental to resolve exacerbations, reduce their episodes and reduce symptoms burden [17].

Some studies have shown that an acute oral administration of salbutamol has an ergogenic effect on sprint exercise in non-asthmatic athletes [42]. Other studies [43,44,45] found that acute salbutamol ingestion during supramaximal or anaerobic exercise improved performance metrics such as peak power output and fatigue resistance and enhanced anaerobic performance during Wingate tests. For the inhaled form and at doses normally prescribed to treat asthma or EIB, systemic effects are negligible and the performance-enhancing potential is low. However, when administered at higher doses, they have greater systemic absorption, and there is evidence that they may enhance sprint performance and muscle strength, especially when given acutely [46]. Despite evidence of ergogenic effects by β_2_-agonists, some doubts still remain and WADA, since the 2008 Games, has made numerous changes to inhaled drugs on the Prohibited List [47].

β-agonists have also systemic metabolic effects, especially on lipolysis, glucose homeostasis, and insulin secretion via β_2_-adrenergic receptors. They act elevating intracellular cAMP, stimulating protein kinase A (PKA) pathways that promote fat breakdown and affect glucose uptake and metabolism [48]. The same pathway accelerates ATP replenishment and promotes a mild anabolic shift by enhancing protein synthesis and suppressing ubiquitin–proteasome–mediated degradation. The net effect can be a modest increase in peak power output during repeated sprints and a faster post-exercise recovery of muscle contractile capacity, though these benefits are dose- and route-dependent [3]. Moreover, inhaled terbutaline enhances anaerobic performance by stimulating glycolysis and increasing power output during repeated sprinting. These effects occur independently of oxygen delivery, suggesting a direct muscle-level effect [49].

## 3. Effects on the Cardiovascular System

### 3.1. Impact of Bronchodilators on Cardiovascular System

Inhaled medications, particularly β_2_-adrenergic agonists such as salbutamol, can exert cardiovascular effects at the molecular level by stimulating β_2_-adrenergic receptors not only in the lungs but also in the heart and vascular smooth muscle. The hemodynamic effects are mediated by β_2_-receptor activation, which increases intracellular cAMP via Gs protein–coupled stimulation of adenylyl cyclase, leading to smooth muscle relaxation and vasodilation. This can cause reflex tachycardia and a mild reduction in blood pressure [20].

Specifically, activation of cardiac β_2_-adrenoceptors results in acute hemodynamic changes such as increased heart rate, increased cardiac output, decreased total peripheral resistance, and reduced parasympathetic outflow [50]. Chronic use of inhaled medications, such as bronchodilators and corticosteroids, is associated with a lower rate of cardiovascular events in people affected by asthma or COPD, due to their anti-inflammatory properties and the reduction in exacerbation events [51].

For what concerns systolic function, a few studies investigated the effects of inhaled medications on heart’s contractility. In patients affected by COPD, a significant increase in RVEF and LVEF has been found at multiple gated radionuclide ventriculography after the administration of salbutamol or pirbuterol [52]. Another study on patients with COPD and lung hyperinflation showed that the combination of indacaterol, a long-acting beta-2 adrenoceptor agonist, and glycopyrronium, a muscarinic antagonist, improved cardiac function by increasing LV end-diastolic volume (EDV) and an increased stroke volume at cardiac magnetic resonance imaging [53]. A recent work on different inhaled beta-2 adrenoceptor agonists has shown a significant increase in LVEF and an improvement in the global longitudinal strain (GLS) after the administration of salbutamol, formoterol and their combination in 24 healthy, non-asthmatic female and male endurance athletes. This effect was particularly relevant in female athletes with a more important increase in LV systolic function than in male athletes. Notably, higher serum concentrations for both salbutamol and formoterol were detectable in female athletes while higher concentrations of salbutamol have been observed with the combined inhalation of salbutamol plus formoterol in male athletes [54].

### 3.2. Potential Cardiovascular Risks Associated with Misuse (e.g., Arrhythmias, Increased Workload)

Use of inhaled medications has also been associated with risks, especially for the cardiovascular system. Side effects of inhaled medications are especially reported when inhaled medications are overused or misused, and this is common in healthy athletes using this class of drug to enhance their physical performance [55]. Salbutamol, for example, has been demonstrated to cause a reduction in vascular function and an increase in arterial stiffness in people suffering from asthma [56]. The potential risk of side cardiac effects with abuse of beta-2 adrenoceptor agonists is linked to their action on β_1_- and β_2_-adrenoceptors, both expressed in the heart tissue and involved in positive cardiotonic chronotropic regulation, cardiac myocyte growth and cardiac toxicity [57]. As previous mentioned, the shift in cardiovascular autonomic control towards decreased parasympathetic tone associated with the acute use of salbutamol could lead to an increase in heart rate and cardiac output with an overstress of the heart [50]. In fact, some of the most common adverse side effects are tachycardia, supraventricular and ventricular arrhythmias, hypokalemia, gastrointestinal disturbances or tremor [58,59].

Moreover, beta-2 adrenergic agonists such as salbutamol and albuterol, have a well-documented effect on serum potassium levels. Through beta-2 receptors stimulation on cell membranes, they activate the sodium-potassium ATPase pump and driving potassium from the extracellular space into cells. This mechanism can lead to transient hypokalemia, especially when beta-2 agonists are used in high doses or administered repeatedly, such as during acute asthma exacerbations or when used via nebulization, augmenting the risk of arrhythmias [60]. Studies have also demonstrated that beta-2 agonists can prolong the corrected QT interval (QTc), in some cases unmasking congenital long QT syndromes in previous asymptomatic patients [61]. Terbutaline, for example, has shown to prolong QTc in young and healthy male subjects; this effect was closely associated with a decrease in plasma potassium levels, augmenting the risk for malignant arrhythmias [62].

Inhaled anticholinergics, such as ipratropium and tiotropium, have also been scrutinized for potential cardiac risks. These drugs can reduce parasympathetic activity and may lead to increased heart rate and reduced heart rate variability, which are markers of cardiac autonomic imbalance [63]. Tiotropium, in particular, has raised concerns due to early reports suggesting an elevated risk of stroke and myocardial infarction; however, subsequent large-scale trials such as the TIOSPIR study did not confirm these findings, and tiotropium is now generally considered safe for most patients when used as directed [64,65].

Theophylline’s cardiovascular effects warrant careful consideration. In healthy individuals, theophylline can increase heart rate and cardiac output, effects that are more pronounced during physical exertion or in combination with beta-agonists [66]. In patients with congestive heart failure, theophylline may enhance sympathetic activity and improve ventilation without significantly worsening hemodynamics [67]. Moreover, elevated plasma concentrations are associated with significant arrhythmic risk, including both supraventricular and ventricular arrhythmias, which can occur even in the absence of preexisting cardiac disease [68].

Inhaled corticosteroids may also pose cardiovascular risks when used in high doses or over long durations. Although systemic absorption is lower compared to oral corticosteroids, chronic ICS use has been associated with metabolic changes, including hypertension, dyslipidaemia, and insulin resistance, which are all risk factors for cardiovascular disease [69]. Table 1 summarizes the main cardiovascular effects and associated risks of different classes of inhaled medications, highlighting their impact on cardiac function and potential implications for athlete health and doping considerations.

## 4. Effects on Muscle Function

### 4.1. Influence on Muscle Contraction and Performance

The effect of β_2_-agonists on skeletal muscle contraction and hypertrophy is well established. This occurs through β_2_-receptor stimulation in skeletal muscle, which elevates cAMP and activates protein kinase A (PKA)–mediated signaling [70].

PKA phosphorylates a variety of downstream targets, including CREB (cAMP response element–binding protein) and components of the Akt/mTOR pathway, both of which are involved in promoting muscle protein synthesis and cellular growth [71,72]. Additionally, PKA activation modulates eukaryotic elongation factor 2 (eEF2) and Akt2, enhancing translation and anabolic signalling [71]. This cascade supports fiber-type remodeling (favoring fast-twitch MHC IIa fibers), increased myofibrillar protein synthesis, and net muscle hypertrophy, especially when combined with resistance training [73]. However, the effects appear to vary by training type, present during habitual and resistance training, but absent during endurance training [74].

Acute administration of high doses of terbutaline in trained men have shown to improve Ca^2+^ release from the sarcoplasmic reticulum, leading to increased contractile force and power output during maximal voluntary and evoked contractions. Moreover, it counteracted exercise-induced reductions in Na^+^–K^+^-ATPase, preventing impairing ion balance and muscle excitability [75]. Oral administration of terbutaline has also shown to produce an increase in lean muscle mass and insulin sensitivity in healthy young men. This effect is not accompanied by canonical increases in GLUT4 or metabolic enzyme content, implying that muscle hypertrophy itself is a major contributor to improved glucose disposal [72]. Also salbutamol may enhance calcium sensitivity in muscle fibers, amplifying the benefits of resistance exercise. Combining oral salbutamol with targeted resistance training effectively maintains, and even improves, ankle extensor strength during prolonged unloading, particularly benefiting women [76]. This evidence suggests that β_2_-agonist administration may be a viable countermeasure against disuse-induced muscle loss, but it could also raise concerns about doping, particularly in strength sports.

Although most mechanistic evidence derives from studies using systemic or oral β_2_-agonist administration, comparable effects on intracellular signalling, muscle contractility, and performance have also been observed following inhaled administration, particularly at high therapeutic or supratherapeutic doses of terbutaline, formoterol, and salbutamol.

High-dose inhaled terbutaline has proven to enhance muscle strength, sprint performance and lean body mass in trained men [77]. However, prolonged use of terbutaline has been shown to hinder skeletal muscle adaptation in response to high-intensity training [71]. Formoterol and salbutamol can acutely increase muscle strength and power output in healthy individuals and trained athletes [78,79]. The mechanism involves the same β_2_-receptor-mediated modulation of intracellular signalling pathways previously discussed that enhance calcium handling, sodium-potassium pump activity, and sarcoplasmic reticulum function, factors critical for rapid and forceful muscle contractions [80]. The increased glycolytic flux and anaerobic energy production during sprinting, potentially delaying the onset of fatigue and supporting higher power outputs [49,78]. Chronic administration may further amplify these effects by promoting muscle hypertrophy and fiber-type shifts toward more glycolytic phenotypes [81].

Use of GCs, particularly oral or injectable forms, was associated with improvements in endurance-related outcomes such as time to exhaustion and exercise capacity in healthy individuals. They may lead to moderate improvements in submaximal and maximal exercise performance, likely due to their anti-inflammatory, euphoric, and metabolic effects [82,83]. However, there is currently no clear evidence regarding the effects of inhaled corticosteroids (ICS) on these outcomes.

### 4.2. Potential Benefits for Strength and Power Athletes

The ergogenic potential of inhaled β_2_-agonists has gained significant interest in strength and power disciplines, where short bursts of high-intensity performance are crucial. Though traditionally used for bronchodilation in asthmatics, evidence suggests that β_2_-agonists, particularly in supratherapeutic doses or under specific conditions, may improve anaerobic performance, muscle strength, and power output in non-asthmatic individuals [10,84,85].

Meta-analyses and controlled trials confirm that inhaled β_2_-agonists like salbutamol and formoterol can produce acute improvements in maximal voluntary contraction, sprint performance and muscle power, albeit with individual variability [10,39]. For example, formoterol inhalation has been shown to significantly enhance repeated sprint ability and high-intensity cycling performance in elite athletes, likely by increasing muscle contractility, reducing fatigue, and improving glycolytic energy contribution [78,86].

Mechanistically, these benefits are largely attributed to β_2_-receptor-mediated pathways that enhance calcium handling, improve sarcoplasmic reticulum function, and increase Na^+^–K^+^-ATPase activity, facilitating faster and more forceful contractions during high-intensity efforts [78]. Despite these findings, the magnitude of ergogenic effects appears dose-dependent and context-specific. Standard therapeutic doses of inhaled salbutamol (e.g., ≤800 µg/day) have generally shown limited or no significant impact on muscle strength or sprint performance in non-asthmatic athletes [87,88,89]. However, higher doses (exceeding WADA’s permitted limits) can lead to measurable increases in lean mass, muscle force, and sprint capacity, raising concerns about doping and unfair advantages in competitive environments [90,91]. However, chronic use of terbutaline has been reported to impair skeletal muscle adaption following high-intensity training [71].

While β_2_-agonists have shown ergogenic potential, the effects of corticosteroids on strength and power performance appear to differ depending on the route of administration. Oral corticosteroids, such as prednisolone, have demonstrated some capacity to improve endurance and high-intensity exercise performance when used acutely or over short periods. Several studies involving acute or short-term oral prednisolone intake reported improved time to exhaustion, enhanced endurance capacity, and altered substrate utilization during submaximal exercise. These effects are thought to result from glucocorticoid-induced euphoria, blunting of fatigue perception, anti-inflammatory effects, and shifts in energy metabolism [92,93,94]. In contrast, inhaled corticosteroids (ICS) appear to have limited or no ergogenic effects on strength and power performance in healthy athletes. Kuipers et al. (2008) found that four weeks of inhaled corticosteroid treatment did not enhance maximal power output in trained endurance athletes [95]. While ICS may reduce airway inflammation and improve pulmonary function in asthmatic athletes, their systemic absorption is minimal, and their effects on muscle performance are negligible in healthy subjects. Table 2 summarizes the mechanisms, muscle effects, and performance outcomes of inhaled and oral β_2_-agonists and corticosteroids.

## 5. Doping Concerns and Ethical Implications

### 5.1. Current Regulations and Guidelines on Permissible Thresholds for Therapeutic Use

Inhaled medications occupy a complex regulatory space because they are both essential treatments and potential performance enhancers. As previously discussed, the ergogenic effect depends on formulation, dosage, and type of activity. At standard therapeutic inhaled doses, ergogenic effects are minimal, but high doses improve anaerobic power and muscle strength.

To mitigate the potential misuse of inhaled medications while ensuring access for athletes with legitimate medical needs, WADA has established regulatory thresholds that reflect a nuanced approach that differentiates between therapeutic use and potential misuse. All β_2_-agonists are classified as prohibited substances under Section S3 of the 2025 WADA Prohibited List, applicable both in- and out-of-competition [96]. This category encompasses both selective and non-selective β_2_-agonists, including substances such as salbutamol, salmeterol, formoterol and vilanterol. The rationale for this classification stems from the potential performance-enhancing effects of these drugs, which, when used improperly, can provide athletes with an unfair advantage. However, WADA acknowledges the legitimate medical use of certain inhaled β_2_-agonists at established therapeutic doses for conditions like asthma and EIB, leading to specific exceptions in the regulations.

For inhaled salbutamol, WADA permits a maximum dose of 1600 micrograms over 24 h, with no single dose exceeding 600 micrograms within any 8-h period. Similarly, inhaled formoterol is allowed up to a maximum delivered dose of 54 micrograms over 24 h, with no more than 36 micrograms administered within any 12-h period. Inhaled salmeterol is permitted up to 200 micrograms per 24 h, and inhaled vilanterol up to 25 micrograms per 24 h. These dosage limits are designed to ensure that the use of these medications remains within therapeutic boundaries and does not confer performance-enhancing benefits.

A critical aspect of WADA’s regulations is the monitoring of urinary concentrations of these substances. The presence of salbutamol in urine exceeding 1000 ng/mL or formoterol exceeding 40 ng/mL is considered inconsistent with therapeutic use and will be regarded as an Adverse Analytical Finding (AAF), potentially leading to an anti-doping rule violation (ADRV) (Prohibited List 2026) and a period of ineligibility from sport or the potential stripping of all results from the date of the violation [9]. In such cases, the athlete bears the burden of proving, through a controlled pharmacokinetic study, that the elevated levels resulted from the permitted inhaled doses. Recent data on AAFs for commonly misused β_2_-agonists highlight trends in anti-doping testing. In 2022, testing identified 47 cases of terbutaline, 7 cases of salbutamol, 2 cases of fenoterol, and 154 cases of clenbuterol [97]. In 2023, terbutaline was detected in 35 cases, salbutamol in 15 cases (approximately twice the number reported in 2022), fenoterol in 5 cases (also a two-fold increase), and clenbuterol in 34 cases, representing a substantial decrease compared with 2022 [98]. These figures illustrate ongoing monitoring of inhaled β_2_-agonists and clenbuterol in professional sports and underscore fluctuations in detected AAFs over time. Clenbuterol is a β_2_-agonist with bronchodilator activity that is approved for human use in a limited number of countries (e.g., parts of Eastern Europe and Latin America) for conditions such as asthma or COPD, but it is not approved in the United States of America and in Europe. The notably high numbers of AAFs for clenbuterol may reflect several overlapping factors: (1) intentional doping use, exploiting its combined anabolic, fat-loss, and bronchodilation properties; (2) therapeutic use without an appropriate Therapeutic Use Exemption (TUE) in those jurisdictions where it is legitimately approved; (3) unintentional ingestion from contaminated meat or animal products, especially in countries where clenbuterol is used illegally in livestock, which has been documented as a source of inadvertent positive tests. Finally, contaminated dietary supplements (often fat-burner products or illicit preparations) may also contribute to inadvertent findings [99].

Athletes requiring the use of inhaled β_2_-agonists beyond the specified limits must apply for a TUE. The TUE process involves a thorough review of the athlete’s medical history, diagnostic tests (such as spirometry or bronchoprovocation tests), and justification for the need to exceed standard dosage limits. This process ensures that the use of these medications is medically necessary and does not provide the athlete with a competitive advantage. The TUE system is integral to balancing the legitimate medical needs of athletes with the overarching goal of preserving the integrity of competition.

ICS, conversely, are not prohibited by WADA when administered via inhalation at doses within the manufacturer’s recommended limits. This includes medications such as fluticasone, budesonide, and beclomethasone.

Despite its safeguards, the TUE system has faced criticism for potential inconsistencies, lack of transparency, and the possibility of abuse, especially when some national anti-doping organizations appear more lenient or rigorous than others in approving exemptions. Moreover, distrust in TUE administration has been reported among athletes, especially in those that have had experience of TUEs [100].

### 5.2. Considerations in Medical Use Versus Doping Misuse

Several high-profile doping cases highlight the misuse of inhaled medications. In 2017, a British cyclist tested positive for salbutamol above permitted levels during the Vuelta a España. A year later, UCI and WADA accepted that the elevated concentration could be explained by a severe asthma attack, not intentional misuse, and closed the case [12]. In 2014, an Italian cyclist exceeded the urinary threshold for salbutamol. The Swiss Olympic Disciplinary Chamber concluded he had acted negligently but without intent to dope, resulting in a 9-month suspension [13]. Another case involved a Spanish cyclist who received a 2-year ban and was stripped of his 2010 Tour de France and 2011 Giro d’Italia titles after testing positive for clenbuterol, a β_2_-agonist with anabolic effects used off-label as a bronchodilator [14]. More recently, in 2020, a Swiss cyclist Schelling tested positive for the β_2_-agonist terbutaline during the Tour du Rwanda and received a four-month ban for a non-intentional anti-doping rule violation, as terbutaline use requires a valid TUE [101].

The distinction between therapeutic use and performance-enhancing misuse is a central ethical concern in sports medicine. As previous exposed, athletes have a higher prevalence of asthma and EIB compared to the general population. While this could reflect better diagnostic vigilance and environmental exposure (e.g., to cold air, chlorine, or pollutants), some experts argue that diagnostic inflation may be occurring, where athletes without clinical need are prescribed inhaled medications to gain a competitive advantage [102].

This concern is compounded by the potential ergogenic effects of β_2_-agonists in supratherapeutic doses that suggests that even marginal misuse can shift the balance of competition, violating the ethical principles of fairness and respect for opponents.

On the other hand, denying access to needed medications out of suspicion or over-regulation can harm athletes with genuine medical conditions. The perception that asthma medication may enhance sports performance has created a negative stigma towards athletes with asthma, inhaler therapy, and TUEs. In this context, both athletes and clinicians may have inadequate knowledge of current anti-doping policies regarding asthma medication, increasing the risk of receiving AAFs, committing ADRVs, or undertreating asthma [100,103]. Poorly controlled asthma can severely impair performance and increase the risk of exercise-related complications such as airway collapse, hypoxemia, or even hospitalization [104]. Moreover, it has been suggested that stigma surrounding asthma treatment and concerns about violating anti-doping rules may be leading some athletes to avoid adhering to their prescribed therapy [105]. Therefore, ethical policymaking must protect both the athlete’s health and the integrity of sport by facilitating timely treatment while implementing rigorous oversight mechanisms to detect abuse.

The challenge lies in identifying and closing the regulatory grey zones. Recent advancements such as the Athlete Biological Passport (ABP), which tracks individual physiological parameters over time, may offer promising tools to detect unusual patterns indicative of misuse, along with “omics” strategies and artificial intelligence [106,107]. Continued research into drug metabolism, objective diagnostic thresholds, and individualized response profiles may also support more accurate assessments of legitimate need versus strategic use. Moreover, promoting education for athletes, coaches, and healthcare providers on ethical medication use and the potential risk of doping is critical in fostering a culture of integrity and responsibility.

**Figure 1:**
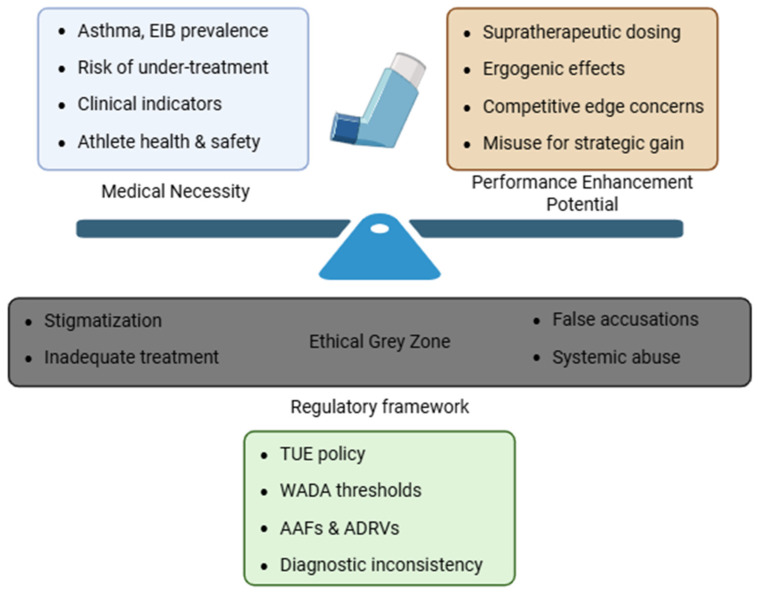
Ethical Complexity Surrounding inhaled medication use in sport. The image highlights the ethical and regulatory challenges in managing asthma treatment in athletes, weighting clinical need against potential performance enhancement. An ethical grey zone emerges, consisting of stigmatization, risk of undertreatment, false accusations and systemic abuse. In this context, the regulatory framework, based on WADA policies, aims to mediate this complex landscape.

Addressing these challenges requires a combination of science-based policy, transparent governance, and a shared commitment to both athlete health and fair play. Figure 1 illustrates the ethical tensions surrounding inhaled medication use in sport.

## 6. Detection Methods for Inhaled Medications

### 6.1. Urine and Blood Testing Protocols

The primary matrix for monitoring inhaled β_2_-agonists in athletes is urine, owing to its non-invasive collection and extended detection window. Blood sampling, while more specific for pharmacokinetic profiling, remains less practical due to rapid clearance and logistical challenges in anti-doping settings. The WADA permits therapeutic inhalation of β_2_-agonists such as salbutamol and formoterol within specified dose limits yet enforces urinary thresholds to discriminate acceptable use from doping violations. The limit for salbutamol in urine, for example, is 1000 ng/mL, while formoterol has a threshold of 40 ng/mL [96]. The regulatory framework is codified in WADA’s Technical Document TD2022DL, which details decision limits (DLs), internal standardization, chromatographic confirmation, ion-ratio verification, and minimum performance criteria [108].

Urinary thresholds are designed to accommodate typical therapeutic dosing while flagging supratherapeutic usage. However, the practical application of these thresholds is complex due to large inter-individual and intra-individual variability. Empirical findings confirm that inhaled formoterol and salmeterol yield urinary concentrations in the low to sub-ng/mL range following therapeutic inhalation, comfortably below WADA thresholds for formoterol and far below salbutamol’s limit [109,110].

However, numerous studies have reported that urinary concentrations of inhaled β_2_-agonists can vary significantly due to factors beyond dose. Haase et al. (2016) documented that intense exercise and moderate dehydration markedly elevated urinary salbutamol levels following high-dose administration, calling into question the reliability of thresholds in athletic contexts [111]. Similarly, Dickinson et al. (2014) demonstrated differences in excretion linked hydration status, irrespective of gender or ethnicity [112]. Both findings emphasize the need for context-specific judgment when interpreting urinary results. Moreover, Heuberger et al. (2018) critically highlighted the futility of current urine-based detection protocols for salbutamol [113]. Their analysis concluded that the high threshold and wide variability contribute to frequent false negatives; athletes taking supratherapeutic doses may remain below the set limit, while therapeutic usage under burdening conditions could trigger false positives. This raises serious concerns about the fairness and reliability of urine-based control strategies [113].

Blood or plasma sampling can offer higher resolution snapshots of recent drug intake, reflecting peak concentrations more faithfully than urine. However, β_2_-agonists exhibit rapid absorption and clearance from plasma, meaning that sampling must occur within hours after dosing [114]. Practical challenges such as multiple sampling needs, invasive collection, and ethical considerations limit adoption in competition settings, relegating blood testing to clinical studies or investigatory follow-ups. Despite these limitations, combining plasma with urine can help pinpoint timing and dosing.

For salmeterol, whose use is also permitted via inhalation, no formal urinary threshold is currently defined by WADA. However, Jacobson et al. (2017) used enantioselective UPLC-MS/MS to quantify (R)- and (S)-salmeterol in urine after single doses of 50 µg and 200 µg [115]. They reported median peak concentrations of 0.084 ng/mL and 2.1 ng/mL respectively, with a maximum observed level of only 5.7 ng/mL. These findings suggest that inhaled racemic salmeterol results in urinary concentrations well below typical analytical cut-offs, highlighting the critical need for more sensitive assays and/or threshold guidelines [115].

### 6.2. Advanced Technologies for Detecting Bronchodilators

The evolution of High-Resolution Mass Spectrometry (HRMS) platforms empowers simultaneous screening of multiple prohibited substances with unmatched sensitivity. Liu et al. (2023) employed an HRMS-based workflow capable of co-detecting an array of β_2_-agonists, their metabolites, and conjugates in a single sample [116]. This approach significantly reduces false negatives from unrelated drug presence or metabolic cross-interference [116]. Similarly, Thevis et al. (2022) lauded non-targeted HRMS profiling as a transformative tool, capable of identifying unknown metabolites, structural analogues, and emerging doping agents, including designer β_2_-agonists [117]. High-resolution data can retrospectively identify new analytes upon database updates, extending the lifespan of each sample and strengthening retrospective analysis [117].

Chiral separation techniques afford precise targeting of enantiomers such as (R)- and (S)-salmeterol, which possess divergent pharmacodynamics and metabolic rates. Jacobson et al. (2022) demonstrated that measuring the R-enantiomer improves both sensitivity to recent therapeutic use and specificity, making differentiations between metabolic sources [118]. The “salmeterol anomaly” further highlights cases in which nonspecific methods flagged samples that, under enantioselective scrutiny, aligned with therapeutic administration [118].

Metabolite-targeted assays seek to detect both parent β_2_-agonists and their phase II conjugates or phase I oxidized products. Orlovius et al. (2009) discovered a sulfoconjugated metabolite of terbutaline, a marker absent in the base drug, into doping detection tools [119]. Chundela & Große (2015) developed LC–MS/MS assays to detect vilanterol and olodaterol, including their specific metabolites, enabling detection in low-dose therapeutic contexts [120].

While mass spectrometry is central to laboratory testing, immunoassay and biosensor methods are under development for rapid, high-throughput screening. Ouyang et al. (2022) reviewed immunoassays capable of detecting β_2_-agonists across foods and biologics, highlighting their low-cost, user-friendly potential [121]. Xu et al. (2022) introduced a colloidal gold-based immunosensor capable of detecting up to 12 β_2_-agonists simultaneously in urine with high sensitivity [122]. Though not yet accepted in WADA labs, such tools may streamline preliminary rounds of testing. These methods offer portability and speed, important advantages for in-competition screens or field testing. Limitations include cross-reactivity issues and generally lower specificity compared to MS-based confirmatory methods, which require ongoing refinement to meet WADA accuracy criteria.

Recently, the microsampling technique of dried blood spots (DBS) has emerged as a promising complementary matrix to urine and conventional venous blood in anti-doping control. WADA has officially approved the use of DBS for doping control through the Technical Document TD2021DBS, which outlines the requirements for sample collection, transport, and analysis [108]. DBS enables minimally invasive collection of micro-volumes of capillary whole blood, offers simplified transport/storage and potentially robust quantitative assessment of circulating drug concentrations, thus opening new perspectives for improved result interpretation [123]. DBS could help bridge the gap between urinary metabolite levels and the actual systemic exposure of an athlete, supporting more accurate interpretation of quantitative results in anti-doping investigations.

### 6.3. Challenges in Detecting Inhaled Medications Due to Short Half-Lives and Dosage Variation

Short elimination half-lives, typically in the range of few hours, characterize many inhaled bronchodilators. In athletes undergoing intense physical training, both salmeterol and its α-hydroxymetabolite fall below quantifiable limits within 12–24 h after a single dry powder inhalation. In contrast, chronic inhalation may lead to higher concentrations of both the drug and its metabolite in the bloodstream compared to a single dose [124]. Similarly, urinary profiles of vilanterol and its metabolites, following both therapeutic and elevated dosing, highlight the rapid decline and significant inter-individual variability of both the parent compound and its metabolites [125]. These findings underscore the risk of false-negative results when sampling does not occur near peak excretion.

Urinary drug concentrations do not scale predictably with inhaled dose due to influences such as device aerosol characteristics, pulmonary deposition, swallow/absorption ratios, and metabolic pathway activation. Fitch (2018) critiques the assumption that urine concentration equates dose, emphasizing that route and method critically skew interpretations [126]. Heuberger et al. (2018) further demonstrated that toxicologically significant doses could remain undetected in standard control regimes [113].

Terbutaline’s case supports this view. Jacobson and Hostrup (2017) advocated for introducing a dose-based urinary threshold for inhaled terbutaline, arguing that clearly defined dose-concentration relationships would help distinguish therapeutic use from misuse, reduce false positives, and align anti-doping policy with pharmacological evidence [127].

Athletic activity, particularly endurance sports, affects pharmacokinetics, notably through dehydration, redistribution of body fluids, and increased excretion. Haase et al. (2016) confirmed that 1600 µg salbutamol inhaled post-exercise led to urinary concentrations exceeding 2000 ng/mL in dehydrated subjects, twice WADA thresholds [111]. Similarly, Dickinson et al. (2014) showed hydration and activity independently influence detection, implicating technical thresholds in risking false enhancement or suppression of results [112].

Detection protocols must address both parent compounds and their conjugates. For instance, terbutaline sulfoconjugates may persist in detectable form even when the base drug has cleared. Accurate detection relies on validated assays to include both classes. [119].

The proliferation of new inhaled drugs (e.g., olodaterol, vilanterol), introduced for COPD, requires method adaptation. Early detection efforts by Chundela & Große (2015) built assay libraries capable of identifying olodaterol and vilanterol, showing imperative response times required for emerging inhalants [120].

Many β_2_-agonists are chiral, and their enantiomers often differ in both pharmacological activity and metabolic fate. Standard immunoassays and non-chiral LC-MS methods cannot distinguish between these enantiomers. For example, racemic salmeterol yields a mixture in urine, though only the (R)-enantiomer is pharmacologically active. Jacobson et al. (2022) demonstrated that enantioselective measurement of urinary salmeterol offers improved interpretive clarity and may support future doping control efforts, especially if thresholds are introduced [118]. Their findings highlight the analytical advantage of resolving enantiomer-specific disposition in evaluating therapeutic use versus potential misuse [118].

WADA’s guidelines provide crucial structure but depend on the integrity of confirmatory tests [108]. Invalid or borderline results may unjustly penalize athletes or, conversely, fail to identify substance abuse. Addressing this challenge requires robust cross-laboratory validation of HRMS, immunoassay screens, and emerging analytical platforms, alongside inter-laboratory ring trials to ensure jurisdictional consistency. Furthermore, data-driven adjustments to urinary thresholds, particularly for chiral drugs or compounds with unpredictable metabolite profiles, are essential to improving both fairness and analytical accuracy. Increasingly, contaminated over-the-counter products and dietary supplements introduce β_2_-agonists unintentionally. For example, higenamine is a plant-derived beta-2 adrenergic agonist found in various herbal supplements, known for its stimulant and fat-burning properties, that is banned by WADA at all times due to its potential performance-enhancing effects. Stojanovic et al. tracked urinary higenamine in women following supplement consumption, showing potential interference with doping tests [128]. Thorough education on all relevant sources, plus careful laboratory source verification, is essential to avoid accidental doping cases.

Non-targeted metabolomic profiling detects secondary metabolites and metabolic fingerprint shifts characteristic of bronchodilator use, often persisting after the parent drug has been cleared. Kiss et al. applied ultra-HRMS paired with multivariate statistics to urine from athletes who used salbutamol or budesonide [129]. They identified distinct metabolite signals, beyond the parent compounds, that differentiated treated individuals from clean controls, demonstrating how HRMS-derived metabolic patterns can reveal drug exposure even when standard assays no longer detect the drug. Similarly, Coll et al. systematically characterized the urinary excretion profiles of budesonide and its metabolites after varying routes of administration (intranasal, inhaled, and oral), emphasizing that metabolite patterns differed significantly depending on dose and delivery method [130]. These findings underscore the value of incorporating detailed metabolite mapping into anti-doping protocols, not only for performance-enhancing agents like β_2_-agonists, but also for substances like inhaled corticosteroids, which may not directly enhance performance yet remain subject to misuse. Integrating metabolomic data could refine threshold interpretations, enable retrospective detection, and improve fairness by reducing both false positives and undetected infractions.

## 7. Conclusions

Inhaled medications occupy a unique position in sports medicine, being essential for the treatment of asthma and EIB; however, they may confer performance benefits when misused. Bronchodilators can enhance cardiovascular function, muscle performance, and recovery at supratherapeutic doses, while inhaled corticosteroids exert mainly anti-inflammatory effects with limited ergogenic potential.

The regulatory framework addresses this duality through WADA thresholds and the TUE system, which provide athletes access to essential therapies for their respiratory conditions while protecting the integrity of competition. Nevertheless, variability in individual responses and detection challenges continue to complicate enforcement, as shown by high-profile cases involving elite athletes and ongoing debates on ergogenic thresholds.

Future progress will rely on more refined detection methods, such as enantioselective assays and metabolomic profiling, together with transparent governance and effective education. Ultimately, ensuring athletes’ health and fair play requires policies that remain scientifically robust, ethically rigorous, and consistently applied.

## Figures and Tables

**Table 1:** Cardiovascular effects and risks associated with different classes of inhaled medications commonly used in athletic populations. CV, cardiovascular; LVEF, left ventricular ejection fraction; RVEF, right ventricular ejection fraction; GLS, global longitudinal strain; LVEDV, left ventricular end-diastolic volume; ICS, inhaled corticosteroids; MI, myocardial infarction; COPD, chronic obstructive pulmonary disease; QTc, corrected QT interval; Na^+^/K^+^ ATPase, sodium-potassium adenosine triphosphatase; ↓ = reduction; ↑ = increase.

Drug Class	Cardiovascular Effects	Cardiovascular Risks
β_2_-Adrenergic Agonists (e.g., Salbutamol, Formoterol, Albuterol, Terbutaline, Indacaterol)	- ↑ Heart rate and cardiac output (acute) - ↓ Total peripheral resistance—↑ LVEF, RVEF, GLS (in COPD and athletes) - ↑ LVEDV and stroke volume (in combo with antimuscarinics)	- Tachycardia, supraventricular/ventricular arrhythmias - ↓ Vascular function, ↑ arterial stiffness - Hypokalemia (due to ↑ Na^+^/K^+^ ATPase activity) - ↑ QTc interval; risk of unmasking long QT - Cardiac stress due to ↓ parasympathetic tone
Muscarinic Antagonists (Anticholinergics) (e.g., Ipratropium, Tiotropium, Glycopyrronium)	- ↑ Stroke volume and LVEDV when combined with β_2_-agonists	- ↑ Heart rate - ↓ Heart rate variability - Autonomic imbalance - Initial concerns about ↑ MI/stroke (not confirmed in large trials)
Inhaled Corticosteroids (ICS) (e.g., beclomethasone, budesonide)	- ↓ CV events in asthma/COPD patients (anti-inflammatory effect)	- Hypertension, dyslipidemia, insulin resistance (with high doses/long-term use) - Potential ↑ long-term CVD risk due to metabolic side effects

**Table 2:** Effects of inhaled and oral β_2_-agonists and corticosteroids on muscle function, performance outcomes, and potential implications for athletes. PKA, protein kinase A; cAMP, cyclic adenosine monophosphate; CREB, cAMP response element–binding protein; mTOR, mechanistic target of rapamycin; eEF2, eukaryotic elongation factor 2; SR, sarcoplasmic reticulum; ICS, inhaled corticosteroids; MHC IIa, myosin heavy chain type IIa; ↓ = reduction; ↑ = increase.

Drug Class	Form & Route	Mechanisms of Action	Observed Effects on Muscle	Performance Outcomes	Context/Limitations
β_2_-Agonists	Oral (e.g., Terbutaline, Salbutamol)	- β_2_-receptor → ↑ cAMP → PKA activation - Akt/mTOR, CREB, eEF2, Akt2 signalling - Fiber-type remodeling (↑MHC IIa) - ↑ Ca^2+^ release, Na^+^–K^+^-ATPase activity	- ↑ Muscle hypertrophy - ↑ Lean mass - ↑ Insulin sensitivity	- ↑ Muscle strength & power - ↑ Sprint performance - Improved glucose disposal	- Greater effect with resistance vs. endurance training - Potential impairment with chronic terbutaline use
β_2_-Agonists	Inhaled (e.g., Terbutaline, Salbutamol, Formoterol)	- Similar signalling as oral form - ↑ Ca^2+^ sensitivity & handling - ↑ Glycolytic flux—↑ Na^+^–K^+^-ATPase & SR function	- ↑ Muscle strength - ↑ Lean body mass - ↑ Fast-twitch fiber recruitment	- ↑ Peak power output - ↑ Sprint & repeated sprint performance - ↑ Anaerobic capacity	- Lower systemic effects vs. oral - High doses needed for notable effect—Mixed findings in non-asthmatics
Corticosteroids	Oral (e.g., Prednisolone)	- Anti-inflammatory—Euphoric effects - Metabolic shift (↑ carb use)	- No direct hypertrophy - Modulation of energy pathways	- ↑ Time to exhaustion - ↑ Endurance capacity - ↓ Fatigue perception	- Effects seen in short-term use - Less data in elite strength athletes
Corticosteroids	Inhaled (ICS)	- Minimal systemic absorption - Anti-inflammatory (local airways)	- Negligible effect on muscle	- No significant ergogenic benefit	- No performance gain in healthy athletes - May benefit asthmatics only

## Data Availability

No new data were created or analyzed in this study.
